# “Duhrssen Scnitt” or Vaginal Cesarean Section in the Treatment of Puerperal Convulsions and the Report of a Successful Case

**Published:** 1906-10

**Authors:** Herman E. Hayd

**Affiliations:** Buffalo, N. Y.


### "Duhrssen Scnitt" or Vaginal Cesarean Section in the Treatment of Puerperal Convulsions and the Report of a Successful Case.

By HERMAN E. HAYD, M. D., M. R. C. S., Eng., Buffalo, N. Y.

THE following instructive case is contributed to the literature of the management of puerperal convulsions.

August 13th, 1906, I was called to see Mrs. Edith K., primipara, aet. twenty-three, who was in convulsions and I found in attendance Drs. Allen and Baker of Williamsville, N. Y. The first convulsion occurred at six A. M., and she had had seven up to two P. M. Large doses of tinct. veratrum viridi had been administered and half grain doses of codeia hypodermically. She had also taken five drops of croton oil by mouth in solution with olive oil.

After cleansing the vagina and giving a bichloride douche, a vaginal examination was made, when it was found that the os was hard, but slightly dilated and admitted one finger, and there was also a show of blood. An attempt was made to forcibly dilate the cervix, but this was at once abandoned, not only because it was impossible to make any impression upon the tissues, but the irritation immediately brought on a terrific convulsion. A second convulsion occurred in about half an hour and it was then decided to remove the patient to the German Deaconess's Hospital for operation. The baby was alive, as was made out by stethescopic examination over the bare skin, and the heart beats being strong, and the term of pregnancy about eight and a half months. While in the ambulance en route to the hospital the patient had two more seizures. She reached the hospital about five-thirty P. M., and was at once taken to the operating room and prepared for operation.

Chloroform was administered, the bladder was emptied and the urine quickly examined, which was found to be nearly solid by the heat and nitric acid test. After shaving and scrubbing the parts thoroughly, the cervix was seized by two volsellum forceps and pulled well down into the vagina, and with a scissors a long anterior incision was made up to the vault. A transverse cut was then made across the roof of the vagina and the bladder and soft parts were quickly separated and pushed well up upon the uterus. Upon passing the finger into the uterus a few fibers of the sharp internal os, or Bandel's ring, remained undivided. These were severed with a blunt bistoury well up into the body of the uterus. There was no hemorrhage. The membranes were then broken and the position of the child accurately determined—left occipto-anterior—the forceps applied and the delivery was slowly and cautiously effected but with considerable force and some difficulty. After the head had engaged in the vagina and was well upon the perineum, the forceps was removed, the head and shoulders were carefully delivered, and the baby—a boy weighing six and three-quarters pounds—was given at once to the assistant, who spent at least thirty minutes in energetic and resourceful efforts at resuscitation.

The placenta was delivered in due time and the cervix was immediately sewed up, as were some tears in the vagina due to the blades of the instrument—and a piece of iodoform gauze was passed up into the uterus through the united cervix. The patient was put to bed in excellent condition. A quart of salt solution was now injected into the loins, in the hope of further flushing the kidneys, and a hypodermic of morphia—¼ grain—was ordered every third hour.

At seven-thirty P. M., she had a convulsion and also a slight one at four-thirty A. M. The next day, August 14th, her condition was excellent; pulse 86, temperature 100°. The morphine was discontinued, and high-up rectal injections of a pint of salt solution were administered every fourth hour. The bladder was emptied by catheter every six hours, and a copious supply of urine was obtained each time. In the evening she began to swallow water and then there was added 1 drachm of a saturated solution of sulphate of magnesia every half hour until free catharsis resulted.

August 15—temperature 99; pulse 72; condition excellent. Mind a little hazy, and taking water freely in large amounts.

August 16—temperature and pulse normal; mind clear. Milk was now administered and baby placed to breast. Gradually a semi-solid diet was given and on the seventeenth day the patient left the hospital well and strong, with the vagina and cervix healed perfectly.

This case demonstrates clearly, first, the wisdom of emptying the uterus as quickly as possible in a bad case of convulsions; and second, the ease and facility with which delivery can be accomplished by employing the anterior cervical incision of Dührssen. I am satisfied that without surgical intervention in this case we should have lost both baby and mother, because the patient had already had ten convulsions before the delivery was brought about, and was a primipara in whom after many hours of waiting and delay, dilatation had only commenced.

493 DELAWARE AVENUE.

